# Integrating Artificial Intelligence and Point-of-Care Ultrasound Within the Clinical-Scientific Method: A Framework for Safer, Smarter Medicine

**DOI:** 10.7759/cureus.106115

**Published:** 2026-03-30

**Authors:** Guillermo Izquierdo-Pretel

**Affiliations:** 1 Hospital Medicine, Jackson Memorial Hospital, Miami, USA; 2 Internal Medicine, Florida International University, Herbert Wertheim College of Medicine, Miami, USA

**Keywords:** artificial intelligence, clinical reasoning, decision support systems, diagnostic safety, medical education, point-of-care ultrasound (pocus), scientific method

## Abstract

This article proposes a novel integrative framework that unifies the clinical method and the scientific method as parallel processes of knowledge generation and decision-making, positioning point-of-care ultrasound (POCUS) and artificial intelligence (AI) as complementary extensions of the clinician’s senses and reasoning that enhance diagnostic accuracy while preserving human judgment. The framework describes how experience and evidence interact through observation, hypothesis generation, testing, and revision, and illustrates how POCUS improves bedside diagnostic precision while AI supports data integration and interpretation. The central takeaway is that integrating AI and POCUS within the clinical-scientific method enables more structured interpretation of clinical data, reduces diagnostic uncertainty, and supports more timely and accurate decision-making while preserving clinician judgment as central.

## Introduction and background

Modern medicine is undergoing a profound transformation driven by the rapid expansion of digital technologies, particularly artificial intelligence (AI), advanced diagnostic imaging, and point-of-care ultrasound (POCUS). AI refers to computational systems capable of performing tasks that typically require human intelligence, such as pattern recognition and decision support. A major component of AI is machine learning, in which algorithms learn from data to improve performance over time. POCUS is the use of portable ultrasound at the bedside to provide real-time diagnostic information during clinical evaluation.

These tools promise earlier detection of disease, greater diagnostic precision, and more efficient clinical workflows. At the same time, they introduce new challenges. Physicians now face an unprecedented volume of clinical data, ranging from electronic health records and imaging studies to continuous physiologic monitoring and algorithm-generated predictions. The central question, therefore, is not simply whether these technologies will influence clinical practice, but how clinicians can integrate them responsibly into the diagnostic process; this article addresses this question by proposing an integrative framework that aligns the clinical and scientific methods and situates POCUS and AI as complementary tools within structured diagnostic reasoning.

Diagnosis has always been the intellectual core of clinical medicine. The physician’s task is to interpret manifestations of disease (symptoms, physical findings, laboratory values, and imaging results) and translate them into meaningful explanations that guide patient care. This process resembles the structure of scientific inquiry itself: clinicians observe phenomena, generate hypotheses, test those hypotheses through targeted investigations, and revise their conclusions as new information emerges [1,2]. In this sense, the clinical method mirrors the scientific method. Both aim to reduce uncertainty by systematically interpreting evidence.

However, the growing complexity of modern healthcare systems has made diagnostic reasoning increasingly difficult. Clinicians must now navigate large volumes of heterogeneous information, often under time pressure and within complex institutional environments. Evidence-based medicine emerged as one response to this challenge by encouraging the systematic use of research evidence in clinical decision-making [3,4]. Yet even with structured guidelines and statistical models, clinical judgment remains essential because physicians must frequently act under conditions of incomplete information and uncertainty.

Recent advances in AI have introduced powerful tools capable of analyzing large datasets and detecting patterns beyond the limits of unaided human perception. Machine learning algorithms can identify subtle correlations in medical images, predict clinical outcomes, and assist in risk stratification [5-7]. At the same time, bedside technologies such as POCUS have expanded the physician’s sensory capabilities, allowing clinicians to visualize physiological processes in real time during the patient encounter [8]. For example, POCUS can be used at the bedside to rapidly identify pulmonary edema through the detection of B-lines or to assess volume status using venous Doppler patterns such as VExUS. Similarly, AI systems can assist clinicians by analyzing imaging data, predicting clinical deterioration, or summarizing complex clinical information to support decision-making.

Together, these developments raise an important conceptual question: Where do AI and POCUS truly belong within clinical reasoning? In this work, we argue that they are best understood as extensions of the clinical-scientific method, where POCUS augments observation at the bedside and AI supports data integration and interpretation within a structured diagnostic process.

Diagnostic uncertainty remains one of the central challenges of clinical medicine and is a major contributor to diagnostic error and patient harm [9]. Even with advances in biomedical science and imaging technology, the interpretation of clinical data remains complex and imperfect. Studies on patient safety have shown that diagnostic errors continue to occur in modern health systems and may represent a significant source of preventable harm. These errors often arise not from a lack of data, but from difficulty organizing and interpreting the information already available. As healthcare systems generate increasingly large volumes of clinical data, physicians face the paradox of information abundance accompanied by persistent uncertainty. This reality highlights the need for conceptual frameworks that help clinicians integrate experience, evidence, and emerging technologies such as POCUS and AI into a coherent and responsible diagnostic process.

While established models of clinical reasoning and evidence-based medicine provide important foundations [4,10], they do not fully address the integration of real-time imaging and AI-driven data interpretation within a unified diagnostic framework.

This article argues that these technologies should not be viewed as replacements for physician judgment but as instruments that operate within the broader clinical-scientific method. By examining how clinicians observe, interpret, and test information at the bedside, we propose a framework in which experience, evidence, and emerging technologies interact dynamically. Within this framework, AI assists in organizing complex data, while POCUS extends the clinician’s sensory perception. The physician remains central as the interpreter who integrates information, reduces uncertainty, and ultimately makes responsible decisions for patient care.

## Review

Methods

This work is a narrative review and conceptual synthesis based on a selective review of foundational and contemporary literature on clinical reasoning, evidence-based medicine, AI in healthcare, and POCUS. Sources were selected based on conceptual relevance and influence in the field. No predefined search strategy, inclusion criteria, or formal risk of bias assessment were applied, as the objective was theoretical integration rather than systematic evidence synthesis.

The clinical method and the scientific method

Diagnosis as Interpretation of Biological Reality

Modern clinical medicine rests on a fundamental epistemological principle: Disease cannot be observed directly but must be inferred from its manifestations. Since the nineteenth century, physicians have been trained to interpret biological signals emerging from the organism through disciplined observation and structured reasoning. Claude Bernard emphasized that medicine advances when physiological knowledge allows physicians to interpret the meaning of observed phenomena within the living organism [11]. In this sense, the clinician operates not merely as an observer but as an interpreter of biological signals arising from cellular and systemic processes.

The Clinical Method as a Scientific Process

The clinical encounter therefore represents a translation process. Cellular disturbances occur at microscopic levels, yet they manifest through macroscopic signs and symptoms that may be perceived by the physician’s senses or detected through technological extensions of those senses. The traditional physical examination - inspection, palpation, percussion, and auscultation - constitutes the classical framework through which clinicians interpret clinical manifestations, a structure historically formalized in bedside medicine since the time of Laennec and subsequent clinical method development [12].

Diagnosis as Integration of Information and Decision

Diagnosis may be conceptualized as the integration of two fundamental processes: the acquisition of information and the execution of decisions. Every diagnostic act begins with the collection of data (symptoms, physical findings, laboratory results, and imaging observations), which together form the informational substrate from which clinical reasoning emerges. However, the mere accumulation of data does not constitute diagnosis. Diagnosis arises when information is interpreted and translated into decisions regarding the nature of disease and the appropriate course of action [1,2].

Signal and Noise in Clinical Reasoning

Information theory provides a useful framework for understanding this process. Claude Shannon demonstrated that any transmitted signal contains both meaningful information and noise [13]. In clinical medicine, patient data similarly consist of relevant signals - true manifestations of disease - embedded within noise arising from variability, measurement error, and contextual factors. The diagnostic task of the clinician is therefore to distinguish signal from noise, reducing uncertainty through structured observation, hypothesis testing, and integration of multiple data sources.

Diagnostic uncertainty and the limits of data

Diagnostic Error and Patient Safety

The manifestations of disease are embedded within a background of biological variability, measurement error, and contextual complexity. The physician’s task is therefore to distinguish meaningful clinical signals from irrelevant or misleading information. Technologies such as AI and advanced imaging may assist in filtering noise and detecting patterns that would otherwise remain hidden [6,7].

Yet diagnosis cannot be reduced solely to information processing. Clinical practice also requires decision-making under conditions of uncertainty. Physicians frequently must act despite incomplete information, balancing probabilities with ethical responsibilities and practical constraints.

Information Overload and Complexity in Modern Medicine

AI represents a powerful set of computational tools capable of analyzing complex patterns in large datasets. In medicine, AI systems have demonstrated growing potential in areas such as medical imaging, diagnostic prediction, and clinical decision support [5,6]. Machine learning algorithms can identify subtle correlations between variables that may not be immediately apparent to human observers. In domains such as dermatology and radiology, AI systems have already demonstrated performance comparable to expert clinicians in specific tasks [14].

However, the role of AI in clinical medicine extends beyond pattern recognition. One of the most significant contributions of AI may be its ability to organize and structure vast quantities of biomedical information. Modern healthcare systems generate enormous volumes of data through electronic health records, imaging technologies, laboratory diagnostics, and continuous physiological monitoring. Without effective tools for managing this complexity, clinicians risk becoming overwhelmed by increasing volumes of data rather than supported in clinical decision-making, a challenge described in the context of digital medicine and information overload [5,15,16].

Limitations of Evidence-Based Models

Evidence-based medicine has attempted to formalize the use of research evidence in clinical decision making [3,4]. However, the clinician’s judgment remains essential because medical decisions often occur in situations where mathematical certainty is unattainable [4,9].

AI and POCUS in Clinical Practice

AI in Clinical Reasoning

AI, as introduced earlier, refers to computational tools that support clinical decision-making through pattern recognition and data analysis [5,6]. Machine learning algorithms can identify subtle correlations between variables that may not be immediately apparent to human observers. In domains such as dermatology and radiology, AI systems have already demonstrated performance comparable to expert clinicians in specific tasks [14].

However, the role of AI in clinical medicine extends beyond pattern recognition. In this context, AI contributes by transforming complex data into clinically interpretable information that can support reasoning at the bedside. Modern healthcare systems generate enormous volumes of data through electronic health records, imaging technologies, laboratory diagnostics, and continuous physiological monitoring. Without effective tools for managing this complexity, clinicians risk becoming overwhelmed by increasing volumes of data rather than supported in clinical decision-making, a challenge described in the context of digital medicine and information overload [5,15,16].

Nevertheless, the integration of AI into clinical practice must remain aligned with the principles of medical professionalism. Algorithms operate within the limits of the data used to train them, and their outputs must always be interpreted within a broader clinical context. AI should therefore be viewed not as an autonomous decision-maker but as a powerful instrument supporting human clinical reasoning [17].

Within the proposed clinical-scientific framework, the use of AI should be understood as an extension of data processing that supports, but does not replace, clinical reasoning. Effective use of AI requires alignment with core principles of clinical practice, including validation, contextual interpretation, and patient-centered application.

First, AI tools must be critically appraised and validated within the clinical context in which they are applied, as performance may vary across populations and settings [6,17]. Second, AI outputs should be interpreted as supportive information rather than definitive conclusions, requiring integration with clinical findings, including bedside assessment and POCUS [5,12].

Third, clinical decision-making ultimately requires integration within the broader clinical context, particularly in situations characterized by uncertainty or incomplete data, where human judgment remains essential [4,9]. Finally, the implementation of AI should be guided by ethical considerations, ensuring that technological adoption enhances patient care without compromising safety, equity, or trust [17].

These principles are not independent recommendations but are embedded within the broader diagnostic process, where experience, evidence, and technological tools interact to reduce uncertainty and support clinical decision-making.

POCUS as an Extension of the Physical Examination

POCUS enhances bedside assessment by providing real-time visualization of physiological processes during clinical evaluation. Beyond its role as a diagnostic adjunct, POCUS fundamentally alters the structure of clinical reasoning by enabling real-time hypothesis testing at the bedside. In traditional diagnostic pathways, hypotheses are often tested through delayed imaging or laboratory studies, creating temporal separation between observation and verification. In contrast, POCUS allows clinicians to immediately evaluate physiological hypotheses during the patient encounter, transforming diagnosis into an iterative process of observation, testing, and revision.

For example, a clinician evaluating dyspnea may generate competing hypotheses such as pulmonary edema, pneumonia, or volume depletion. Bedside ultrasound enables immediate assessment through detection of B-lines, pleural abnormalities, or venous congestion patterns, allowing rapid confirmation or refutation of these hypotheses. Similarly, cardiac POCUS may reveal unexpected findings-such as intracardiac masses or significant hemodynamic alterations-that were not suspected based on initial clinical evaluation, prompting immediate revision of the diagnostic pathway and management decisions.

In this sense, POCUS operationalizes the clinical-scientific method by collapsing the distance between hypothesis generation and empirical testing, enabling continuous refinement of diagnostic reasoning in real time. This shift redefines bedside evaluation from a predominantly inferential process to one that incorporates direct, dynamic verification of physiological hypotheses.

Complementary Roles of AI and POCUS

AI and POCUS function as complementary tools within the diagnostic process, with POCUS enhancing real-time data acquisition and AI supporting data interpretation. Their integration is best understood within the broader clinical-scientific framework described in the following section.

Integrated clinical-scientific framework of diagnosis

Diagnosis as a Dynamic (Living) Process

Diagnosis can be understood as a dynamic and iterative process in which clinical information is continuously gathered, interpreted, and refined over time. This perspective aligns with established models of clinical reasoning and evidence-based medicine, where knowledge evolves through cycles of observation, hypothesis generation, testing, and revision [4,10].

From this standpoint, diagnosis is not a static label but an evolving construct shaped by the interaction between patient data, clinician experience, and contextual factors. The integration of new tools such as POCUS further enhances this process by providing real-time imaging that augments bedside assessment and supports immediate hypothesis testing.

AI may contribute by organizing complex datasets and identifying patterns not readily apparent to human observers [5,6]. However, the interpretive act-linking biological signals to meaningful clinical decisions-remains dependent on clinician judgment, particularly in complex clinical contexts [9,12].

Thus, diagnosis emerges as a living process that integrates experience, evidence, and evolving technological tools within a structured yet adaptive framework.

The Three Domains: Biology, Observation, Computation

The diagnostic process can be conceptualized as the interaction of three complementary domains: biological reality, clinical observation, and computational analysis. Biological reality refers to the underlying cellular and physiological processes that define disease. Clinical observation represents the manifestations of these processes as perceived through the clinician’s senses and their extensions, including POCUS. Computational analysis encompasses the organization and interpretation of data through analytical tools, including AI.

These domains are not independent; rather, they are linked through a continuous process of information transformation. Biological changes generate observable signals, which are captured through clinical examination and imaging. These signals are then interpreted through reasoning processes that may be supported by computational tools. The clinician acts as the integrator, translating data across these domains into meaningful diagnostic conclusions.

The Diagnostic Cycle: Data, Interpretation, Augmentation, Decision

Within this framework, diagnosis can be understood as an iterative cycle: data acquisition (history, examination, POCUS), signal interpretation (clinical reasoning and pattern recognition), computational augmentation (AI-supported analysis), validation and governance (assessment of data quality, algorithm performance, bias, and contextual appropriateness), and decision-making under uncertainty. The inclusion of validation and governance as a distinct component reflects the need to critically evaluate AI outputs within the clinical context. This includes assessment of model validity, awareness of potential biases, and accountability for decision-making. In this sense, governance is not external to the diagnostic process but embedded within it, ensuring that technological tools are applied safely, transparently, and in alignment with patient-centered care.

Each step contributes to reducing uncertainty by refining the distinction between relevant signal and background noise, consistent with principles derived from information theory.

This model differs from traditional approaches by explicitly incorporating real-time imaging and computational analysis into the diagnostic process as structured components rather than ancillary tools. It also emphasizes that integration occurs through clinician-mediated interpretation, preserving the central role of human judgment.

This framework can be situated in relation to established dual-process theories of clinical reasoning, which distinguish between intuitive (System 1) and analytical (System 2) processes [18,19]. Rather than introducing an independent “System 3,” AI is better understood as a form of computational augmentation that interacts with both systems. AI may enhance System 1 by supporting rapid pattern recognition and assist System 2 by organizing data, quantifying uncertainty, and structuring analytical reasoning. In this sense, AI does not replace clinical cognition but modifies the informational environment within which both intuitive and analytical processes operate, while the clinician remains responsible for integrating outputs into meaningful decisions.

While dual-process theory describes the cognitive mechanisms underlying clinical reasoning, the present framework extends this model by incorporating real-time imaging (POCUS) and computational analysis (AI) as structured components of the diagnostic process, thereby integrating cognition, data acquisition, and data interpretation within a unified clinical-scientific paradigm.

**Figure 1:**
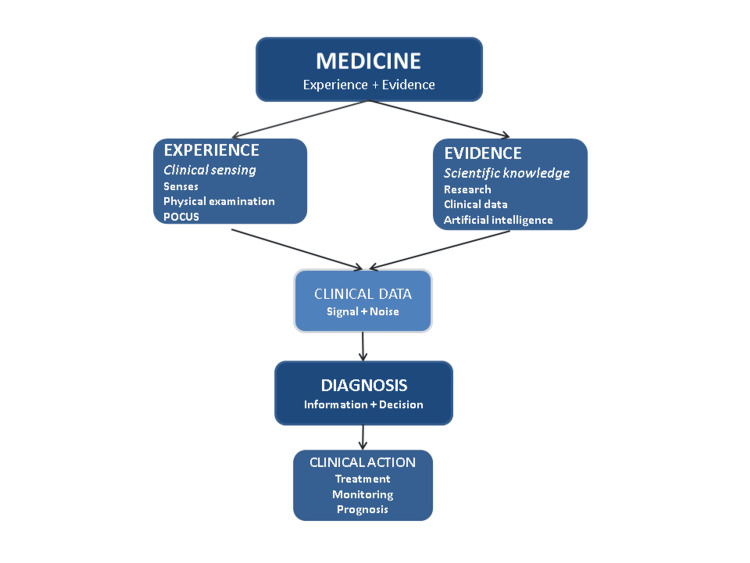
Integrated diagnostic framework linking experience, evidence, POCUS, and AI within the clinical-scientific method Conceptual model illustrating the integration of the clinical and scientific methods, with POCUS extending bedside observation and AI supporting data interpretation within a unified diagnostic process. POCUS: Point-of-care ultrasound; AI: Artificial intelligence

This integrative diagnostic process is illustrated in Figure 1.

Testable Implications of the Framework

The proposed framework generates specific, testable hypotheses that can be evaluated in clinical and educational settings. First, integration of POCUS and AI within structured diagnostic workflows may reduce time to diagnosis, measured as the interval from initial clinical assessment to definitive diagnosis, compared to standard care. Second, the combined use of real-time imaging and AI-supported data interpretation may improve diagnostic accuracy, assessed by concordance with final adjudicated diagnoses or gold-standard testing. Third, this integrated approach may reduce diagnostic error rates, including missed, delayed, or incorrect diagnoses, as defined in patient safety literature. Fourth, incorporation of AI and POCUS into structured clinical reasoning may improve decision consistency and clinician confidence, measurable through interobserver agreement and validated confidence scales. Finally, in educational settings, this framework may enhance learning outcomes, including diagnostic reasoning performance and retention of clinical knowledge. These hypotheses provide a basis for prospective studies designed to evaluate the clinical effectiveness, safety, and educational impact of integrating AI and POCUS within the diagnostic process. These hypotheses also allow for evaluation across different clinical environments, including inpatient, outpatient, and critical care settings, supporting external validity.

Implications for Clinical Practice, Education, and Health Systems

Clinical Practice and Decision-Making

The integration of AI into clinical practice must align with the principles outlined in the proposed framework. Rather than functioning as an independent decision-maker, AI should be applied as a supportive tool that enhances data interpretation within structured clinical reasoning [17]. In practice, this requires that clinicians critically appraise AI outputs, integrate them with bedside findings - including POCUS - and maintain responsibility for final decisions, particularly in conditions of uncertainty. The effective use of AI in clinical care therefore depends not only on technological capability but also on clinician judgment, contextual interpretation, and adherence to patient-centered principles.

Medical Education and Training

The integration of AI and POCUS into clinical practice requires not only technical proficiency but also a strong foundation in medical ethics and professional responsibility. International frameworks emphasize that the use of AI in healthcare must be guided by principles of patient safety, transparency, accountability, and equity [17].

In parallel, the preservation of humanistic clinical care remains essential, as emphasized in discussions on the role of the physician in the age of technology [11]. The clinician’s responsibility extends beyond data interpretation to include contextual judgment, communication, and alignment of decisions with patient values.

Within medical education, this implies that training programs must go beyond technical instruction to include ethical reasoning and critical appraisal of emerging technologies. Clinicians must be prepared to evaluate not only the performance of AI tools but also their appropriate use within the clinical context.

Thus, ethical competence becomes integral to the effective integration of AI and POCUS, ensuring that technological advancements support, rather than undermine, patient-centered care.

Health Systems, Ethics, and Governance

AI can count B-lines, assist with cardiac and VExUS evaluation, segment structures, summarize clinical notes, predict deterioration, or flag risk. In this sense, AI functions as a cognitive stethoscope [5].

However, AI develops inside real healthcare economies. Diagnosis is often tied to billing codes and performance metrics. It is therefore tempting for institutions to prioritize AI systems that improve documentation and revenue rather than AI systems that primarily deepen clinical reasoning. This is understandable, but it becomes risky when economic incentives begin to define the direction of medical innovation [9,15].

AI in healthcare is not limited to a single purpose but operates across a spectrum of applications that include clinical decision support, data integration, workflow optimization, and administrative efficiency. In practice, these functions frequently coexist within the same systems, with tools designed to both enhance clinical understanding and improve operational performance. The relationship between these goals is not inherently oppositional but depends on how technologies are implemented, governed, and aligned with clinical priorities. As a result, the impact of AI on patient care is determined less by its technical capabilities than by the frameworks and values that guide its integration into clinical practice.

Accordingly, the use of AI in clinical practice should be evaluated in terms of its contribution to patient-centered care and its ability to support safe and effective clinical decision-making [9,12].

These considerations highlight that governance is not only an institutional responsibility but also a clinical one, embedded within everyday diagnostic decision-making.

Limitations

This work is a narrative review and conceptual synthesis rather than a systematic review. As such, it does not employ a predefined search strategy, formal inclusion or exclusion criteria, or risk of bias assessment. Consequently, the selection of literature may not fully capture the breadth of available evidence, particularly in rapidly evolving fields such as AI in healthcare. This limitation should be considered when interpreting the scope and generalizability of the proposed framework.

The scope of this manuscript is intentionally focused. It is not intended to provide a technical review of AI, nor to offer detailed discussion of model architectures, prompting strategies, or specialized computational methods. Instead, the objective is to examine the role of AI and POCUS within the clinical method and its parallelism with the scientific method.

Finally, the proposed framework has not yet been empirically validated and should be interpreted as a conceptual model intended to guide future research, education, and clinical integration. Given the rapid evolution of AI, some elements of this framework may require refinement as new evidence, technologies, and regulatory standards emerge. Further studies are needed to evaluate its applicability across different healthcare settings and specialties.

## Conclusions

This article presents an integrative framework that aligns the clinical and scientific methods as parallel processes of knowledge generation, positioning POCUS and AI as complementary tools within diagnostic reasoning. By structuring the diagnostic process as an interaction between observation, interpretation, and decision-making, the framework highlights how these technologies can reduce diagnostic uncertainty and support more accurate and timely clinical decisions while preserving the central role of clinician judgment.

When experience and evidence work together, diagnosis becomes clearer and more reliable. AI can strengthen that process. Economic forces can also distort it. The outcome depends on education, leadership, and ethics. From an ethical perspective, the integration of AI into clinical practice should be guided by the same principles outlined in this framework - namely, validation, contextual interpretation, clinician responsibility, and patient-centered application - ensuring that technological tools support safe, effective, and accountable clinical decision-making.

The implications of this framework extend across clinical practice, medical education, and health system design. For clinicians, the integration of AI and POCUS requires a structured approach to diagnosis that combines data interpretation with contextual judgment, maintaining responsibility for decision-making under uncertainty. For educators, training programs should incorporate not only technical proficiency in ultrasound and familiarity with AI tools but also the development of diagnostic reasoning, critical appraisal skills, and ethical competence. For institutions, the implementation of AI must be guided by validation, transparency, and governance frameworks that ensure these tools are aligned with patient-centered care and clinical safety.

By embedding AI and POCUS within the clinical-scientific method, this framework supports a model of care that is more structured, more informed, and better equipped to manage complexity while preserving the central role of the clinician.
